# Overcharge‐Induced Phase Heterogeneity and Resultant Twin‐Like Layer Deformation in Lithium Cobalt Oxide Cathode for Lithium‐Ion Batteries

**DOI:** 10.1002/advs.202203639

**Published:** 2022-09-11

**Authors:** Juhyun Oh, Seung‐Yong Lee, Hwangsun Kim, Jinseok Ryu, Byeongjun Gil, Jongki Lee, Miyoung Kim

**Affiliations:** ^1^ Department of Materials Science and Engineering and Research Institute of Advanced Materials Seoul National University Seoul 08826 Republic of Korea; ^2^ Division of Materials Science and Engineering Hanyang University Seoul 04763 Republic of Korea; ^3^ Product Engineering Team, Automotive & ESS Biz. Samsung SDI Yongin‐si Gyeonggi‐do 17084 Republic of Korea

**Keywords:** layered lithium transition‐metal oxides, lithium‐ion batteries, mechanical cracks, overcharge, transmission electron microscopy

## Abstract

Overcharging is expected to be one of the solutions to overcome the current energy density limitation of lithium‐ion battery cathodes, which will support the rapid growth of the battery market. However, high‐voltage charging often poses a major safety threat including fatal incendiary incidents, limiting further application. Numerous researches are dedicated to the disadvantages of the overcharging process; nonetheless, the urgent demand for addressing failure mechanisms is still unfulfilled. Herein, it is revealed that overcharging induces phase heterogeneity into layered and cobalt oxide phases, and consequent “twin‐like deformation” in lithium cobalt oxide. The interplay between the uncommon cobalt(III) oxide and the deformation is investigated by revealing the atomistic formation mechanism. Most importantly, abnormal cracking is discovered in the vicinity of the cobalt oxide where structural instability induces substantial contraction. In addition, surface degradation is widely observed in the crack boundary inside the particle. As unintentional overcharging can occur due to local imbalance in state‐of‐charge in severe operating conditions such as fast charging, the issues on overcharging should be emphasized to large extent and this study provides fundamental knowledge of overcharge by elucidating the crack development mechanism of layered cathodes, which is expected to broaden the horizon into high voltage operation.

## Graphical abstract

Overcharged lithium cobalt oxide is investigated in depth to understand the microstructural interplay between the phase heterogeneity and the crack formation during overcharging. Direct observation of the cobalt oxide phases nearby the abnormal crack provides the cracking mechanism of twin‐like deformation. Moreover, the degradation occurring at the crack boundary emphasizes the negative impact of overcharging in relation to physical damages.

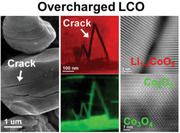

## 1 Introduction

Due to the unique energy‐storage property of the lithium‐ion battery (LIB), the LIB market continues to expand. In addition, with the increasing popularity of electric vehicles, the massive interest in LIBs shows no signs of abating. However, there are major challenges for next‐generation applications, higher energy density, particularly of cathode materials, and safety issues.^[^ ^1^, ^2^ ^]^ Since the discovery of lithium cobalt oxide (LiCoO_2_; LCO) by Goodenough,^[^ ^3^ ^]^ layered lithium transition‐metal oxides, including lithium nickel cobalt manganese oxide (Li(Ni_1−_ *_x_*_−_ *_y_* Co*_x_* Mn*_y_*)O_2_; NCM) and lithium nickel cobalt aluminum oxide (Li(Ni_1−_ *_x_*_−_ *_y_* Co*_x_* Al*_y_*)O_2_; NCA), have been particularly commercially successful as cathode materials. Having an *α*‐NaFeO_2_ (space group R‐3m) type structure, layered‐type cathodes face a common problem, that is, the practical capacity does not match the theoretical capacity.^[^ ^4^, ^5^, ^6^ ^]^ For example, Li_1−_ *_x_* CoO_2_ (0 ≤ *x* < 1) cycles reversibly below the charge cut‐off voltage of 4.2 V (vs Li), which corresponds to an *x* of ≈0.5.^[^ ^3^ ^]^

Intensive researches have focused on expanding the reversibility limit via high‐voltage charging, which inevitably have exposed the drawbacks of so‐called overcharging.^[^ ^6^, ^7^ ^]^ Particularly, the risk of fire and explosion has become a serious concern.^[^ ^8^ ^]^ Overcharging layered‐type cathodes leads to unfavorable oxygen‐ion facing between the transition‐metal oxide slabs. Electrostatic repulsion of anions, arising from the absence of lithium ions, induces phase transformation into spinel or rock salt phases, which is known to impede lithium‐ion reinsertion and thus cause permanent capacity fade.^[^ ^5^, ^6^, ^9^ ^]^ Moreover, overcharging results in the contraction of the interlayer spacing (*d*_0003_), which is suggested to be the cause of mechanical cracks.^[^ ^10^, ^11^ ^]^ A crack acts as an electrolyte permeation pathway and accelerates phase transformation into the interior.^[^ ^12^, ^13^ ^]^ In addition, the high working voltage enhances gas production via exothermic electrolyte decomposition, which can trigger a thermal runaway process leading to ignition.^[^ ^14^, ^15^ ^]^ The importance of overcharging must be understood inclusively concerning local charge heterogeneity. In highly accumulated battery packs, local overcharging of a single cell can pose a risk to the entire cell. Even within a single cell, heterogeneous phase evolution easily occurs in cases such as fast charging.^[^ ^16^, ^17^ ^]^ However, having focused on the negative effects, recent studies mostly emphasized avoiding overcharge and various efforts have been made to alleviate high‐voltage instability.^[^ ^18^, ^19^, ^20^, ^21^ ^]^ Hence, microstructural changes and the mechanism have been overlooked, providing a limited contribution to developing a fundamental solution to overcome overcharging. The ultimate solution could be to develop a cathode material that can support overcharging, therefore, understanding the overcharging mechanism is essential for the realization of a safer next‐generation cathode.

In this work, we investigated both macroscopic and microscopic effects of overcharging in LCO. At charge voltages of 6.0 V (vs Li), catastrophic changes were observed inside and outside LCO particles, even during the first charge cycle. Transmission electron microscopy (TEM) examination of an overcharged sample revealed that overcharging induced phase heterogeneity within the LCO, which consequently led to abnormal crack formation as well as irreversible loss of the lithium‐ion site. This indicates that uneven lithium extraction leads to severe local overcharging, that is, formation of cobalt oxide with substantial lattice contraction resulting in deformation. Thus, the key factor in preventing detrimental effects of overcharging is homogeneous lithium‐ion extraction, which requires a stable self‐standing framework of a transition‐metal oxide without lithium. Since the majority of cathode materials, such as NCM and NCA, share the same structure with LCO, this study improves understanding of the overcharging mechanism of layered‐type cathodes, thus helping to bridge the gap between theoretical and practical capacity.

## 2 Results and Discussion

**Figure 1:**
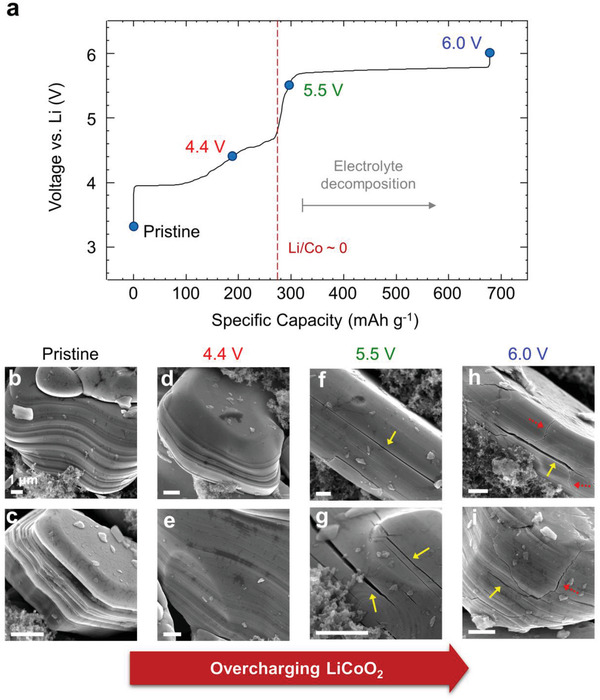
Microcrack development in lithium cobalt oxide (LCO) particles during overcharging. a) Specific capacity as a function of the voltage profile for LCO overcharging. The specific current was 150 mA g^−1^ (1 C‐rate) and the red‐dashed line represents the theoretical capacity limit of LCO. Scanning electron microscopy (SEM) images of b,c) pristine and d,e) 4.4, f,g) 5.5, and h,i) 6.0 V cut‐off samples, obtained under different voltage conditions. Arrows in the SEM images indicate physically damaged LCO particles. Yellow arrows indicate cracks horizontal to the (0003) layer and red‐dotted ones indicate vertical cracks. All scale bars in (b–i) correspond to 1 µm.

To investigate the negative effects of overcharging, four charged states of LCO were compared by varying the cut‐off voltage, that is, pristine and 4.4, 5.5, and 6.0 V‐charged samples. **Figure** **1**a shows the voltage profile of single‐step overcharging to 6.0 V of pristine LCO. The theoretical capacity of LCO is 274 mAh g^−1^, as indicated in Figure 1a by the red dashed line. The 4.4 V cut‐off corresponds to the normal charge condition, while 5.5 and 6.0 V represent two different overcharge conditions. In addition, excess capacity is taken into account based on the electrolyte decomposition in Figure S1, Supporting Information. Figure 1b–i shows scanning electron microscopy (SEM) images of an LCO particle in each stage. Diverse physical damage was observed in the overcharged samples, and microcrack development was evident during the overcharging stages. LCO particles consist of monolithic crystals with a hexagonal shape and stable (0003) plane acting as a flat surface.^[^ ^22^, ^23^ ^]^ Thus, various cracks are classified into two types with respect to the direction of cracking, that is, parallel to the (0003) plane and perpendicular to the (0003) plane. The parallel case (yellow arrow in Figure 1f–i) was observed in both 5.5 and 6.0 V‐charged samples, but the perpendicular case (red‐dashed arrow in Figure 1h,i) was only observed in the 6.0 V‐charged sample. In particular, the former has been reported to appear commonly during cycling,^[^ ^11^, ^12^ ^]^ whereas the latter is reported here for the first time.

**Figure 2:**
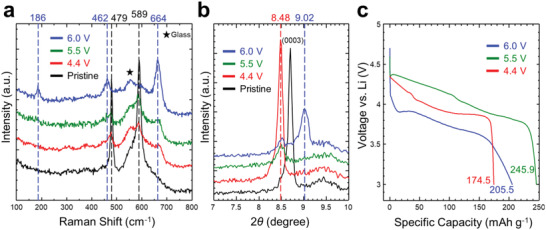
Comparison of lithium cobalt oxide (LCO) among different states of charging. a) Raman spectra of pristine, 4.4, 5.5, and 6.0 V cut‐off samples. Laser excitation of 532 nm radiation was used. Samples were sealed in glass to prevent air exposure. b) X‐ray diffraction plots of pristine and 4.4, 5.5, and 6.0 V cut‐off samples showing the (0003) peak variation. Mo K*α* radiation was used. c) Discharge profiles of LCO after charging to cut‐off voltages of 4.4, 5.5, and 6.0 V. The specific current was 150 mA g^−1^ (1 C‐rate) and the cut‐off voltage for discharge was 3.0 V. Discharging was conducted after a 10 min rest period.

Focusing on the observation that the perpendicular crack was observed only in the 6.0 V‐charged sample, we proceeded with further investigation on each sample in depth. Raman spectra (**Figure** **2**a) confirmed cobalt oxide formation during overcharging; two peaks at 479 and 589 cm^−1^ (corresponding to the E_g_ and A_1g_ modes of LCO, respectively)^[^ ^24^, ^25^ ^]^ decreased in intensity as peaks at 186, 462, and 664 cm^−1^ (assumed as Co_2_O_3_ and Co_3_O_4_)^[^ ^24^, ^25^, ^26^ ^]^ appeared. In the X‐ray diffraction (XRD) patterns (Figure 2b), the main (0003) peak changed markedly. The pristine sample showed a peak at 8.69°, which corresponds to an interlayer spacing of 4.69 Å. After charging to 4.4 V, this peak shifted to 8.48° (4.81 Å), which agrees with the previously reported value of 4.796 Å for Li_0.5_CoO_2_.^[^ ^27^ ^]^ This peak was less intense in the overcharged samples; moreover, the 6.0‐V‐charged sample showed an additional peak at 9.02° (4.52 Å). These values correspond to a lattice mismatch of ≈6%, and support the aforementioned crack formation. The full XRD patterns are provided in Figure S2, Supporting Information. In addition, the sample treated for a long time (10 h) under the condition of 5.5 V was investigated in comparison with other overcharge conditions in Figures S3–S5, Supporting Information. Here increasing the treatment time did not affect the degradation behavior including the formation of cobalt oxide or cracks observed in the 6.0‐V charging sample. Moreover, the 6.0‐V‐charged sample exhibited significant capacity fade and higher polarization in the discharge test (Figure 2c) compared with the 5.5‐V‐charged one. The discharge capacity of the 6.0‐V case was 205.5 mAh g^−1^, whereas that of the 5.5‐V case was 245.9 mAh g^−1^. The differential capacity results of Figure 2c are plotted as a function of charging in Figure S6, Supporting Information. Taken together, the findings indicate that reinsertion of lithium‐ion into the overcharged sample was substantially reduced, which was mainly attributable to irreversible formation of cobalt oxide.

**Figure 3:**
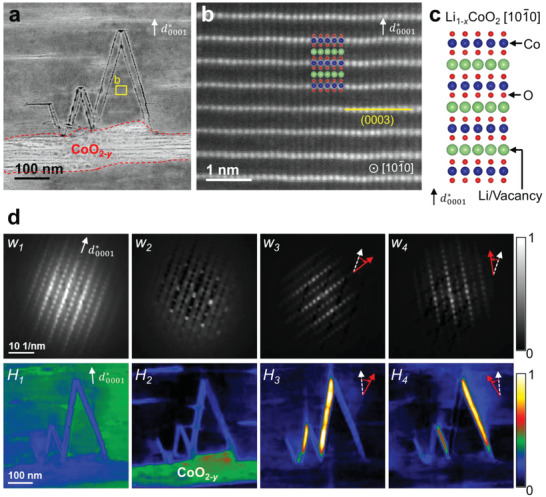
Wedge‐shaped crack at the phase boundary in overcharged lithium cobalt oxide particles. a) High‐angle annular dark‐field (HAADF) scanning transmission electron microscopy (STEM) image of the wedge‐shaped crack. b) Atomic‐resolution HAADF‐STEM image of the region indicated by the yellow box in (a). c) Atomic model of Li_1–_ *_x_* CoO_2_ along the [10‐10] zone axis based on ICDD No. 04‐008‐6329. d) Non‐negative matrix factorization analysis result for the 4D STEM data of the wedge‐shaped crack. The first four components (upper row, *w*_i_) and their corresponding coefficient maps (lower row, *H*_i_) are displayed with normalized intensity. The direction of the reciprocal vector d*_0001_ is indicated in each figure for comparison. The misorientation angle in *w*_3_ and *w*_4_ corresponds to ≈33° and 27°, respectively.

Microscale analysis revealed distinct phase separation in highly overcharged (6.0‐V) LCO (**Figure** **3**). Figure 3a presents a high‐angle annular dark‐field (HAADF) scanning transmission electron microscopy (STEM) image in which mass–thickness contrast reveals partition into brighter and darker phases. Furthermore, an abnormal crack is evident on the darker side, starting from the interface. Intriguingly, the crack has a morphology characteristic of the Sumerian cuneiform; for this reason, it is hereafter referred to as a wedge‐shaped crack. An atomic‐resolution HAADF‐STEM image of the darker phase region is presented in Figure 3b, and confirms the atomic structure as layered R‐3m when viewed along the [10‐10] axis; the atomic model (Li_1−_ *_x_* CoO_2_) is displayed in Figure 3c. In addition, the direction of the reciprocal lattice vector (d*_0001_) is shown, which corresponds to the (0003) plane of the layered structure. To clearly distinguish the unknown phases on the brighter side, scanning electron nanodiffraction patterns were obtained, which are often referred to as 4D STEM patterns. Herein, we employed a dimensionality reduction method, non‐negative matrix factorization (NMF) to extract meaningful information from a multidimensional dataset. NMF was adopted because its non‐negativity constraint accounts for the non‐negative nature of diffraction patterns and offers an intuitive representation of the dataset.^[^ ^28^, ^29^ ^]^ In the case of 4D STEM, factorization yields components (in the form of 2D diffraction patterns) and their corresponding coefficients (representing the 2D spatial distribution of the components) according to a linear‐expression. Figure 3d shows the NMF result for the large 4D STEM dataset composed of 40 000 diffraction patterns. After the factorization, the dataset was expressed as a linear combination consisting of ten components and corresponding coefficients. In other words, the NMF analysis provided ten representative diffraction patterns and their spatial distributions. In the upper row, selected components *w*_i_ (four out of ten) are shown; the corresponding coefficient maps *H*_i_ are shown below each component. The remaining six components are shown in Figure S7, Supporting Information, and a detailed rationale for choosing the number of components in NMF is given in Figures S8–S10, Supporting Information. The first component *w*_1_ represents the overall diffraction pattern; *H*_1_ shows even distribution of intensity while the other components, *w*_2_, *w*_3_, and *w*_4_, represent localized signals. In particular, the green area in *H*_2_ corresponds to the brighter region in Figure 3a. Moreover, the diffraction pattern of *w*_2_ suggests the presence of cobalt oxides, which will be discussed in detail later. Both *w*_3_ and *w*_4_ indicate a misoriented grain of *w*_1_, and at the same time comprise the wedge‐shaped crack. The direction of misorientation of d*_0001_ is indicated by red arrows; the white‐dotted arrow indicates the original direction.

Further analysis of the phase revealed heterogeneity in terms of lithium content. The darker phase in Figure 3a corresponds to the original layered structure, and the electron energy loss spectra in Figure S11, Supporting Information, suggest that there is lithium remaining in this phase. Following the quantification method,^[^ ^30^ ^]^ darker phase shows Li/Co ratios of 0.215 and 0.135, on the other hand, the brighter phase has a Li/Co ratio of 0.020. Thus, the brighter region corresponds to a highly charged state composed of cobalt oxide phases. The atomic density of the different phases is responsible for the mass–thickness contrast in an HAADF‐STEM image. Table S1, Supporting Information, shows the theoretical atomic density of each phase discussed in this study, and Figure S12, Supporting Information, shows corresponding atomic structural models and simulated diffraction patterns. The cobalt oxide phases of Co_3_O_4_ and Co_2_O_3_ are denser structures, in terms of cobalt (*Z* = 27) density, than the original layered structure containing lithium. In other words, the lack of a buffer layer, that is, lithium ions, between the cobalt oxide slabs leads to substantial contraction of the structure, which appears as an area of brighter contrast in an HAADF‐STEM image. Furthermore, the lower‐magnification image in Figure S13, Supporting Information, supports that the residual lithium region is present locally and enclosed by the cobalt oxide phases, which implies how the cobalt oxide phases directly impede lithium extraction.

**Figure 4:**
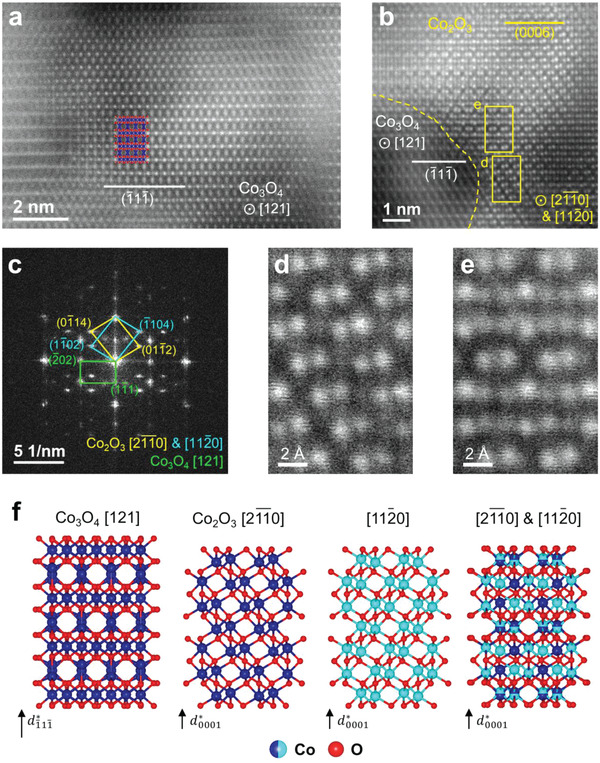
Formation of cobalt oxide phases in the brighter region. Atomic‐resolution high‐angle annular dark‐field scanning transmission electron microscopy (HAADF‐STEM) images of a) the [121] grain of spinel phase Co_3_O_4_, and b) [2‐1‐10] and [11‐20] grains of corundum phase Co_2_O_3_. c) Fast Fourier‐transformation result of (b) showing the crystallographic orientational relationship between cobalt oxide phases. Different colors distinguish different crystal grains. Magnified image of the yellow boxes in (b) highlighting the difference between d) the [2‐1‐10] grain and e) overlapped structure composed of mirror‐symmetric grains [2‐1‐10] and [11‐20]. f) Atomic model of each phase, including the overlapped structure (Co_3_O_4_ ICDD No. 00‐042‐1467 and Co_2_O_3_ ICDD No. 04‐007‐3332).

To understand the cobalt oxide formation, the atomic structure was examined in the brighter region of **Figure** **4**. Spinel Co_3_O_4_ (space group Fd‐3m) and corundum Co_2_O_3_ (space group R‐3c) are present throughout the region. Figure 4a shows an atomic‐resolution HAADF‐STEM image of spinel Co_3_O_4_ along the [121] direction, which is parallel to the [10‐10] direction of the R‐3m structure. This orientational relationship has been reported previously in the spinel phase transformation;^[^ ^12^, ^31^ ^]^ it is attributable to the fact that both phases share a face‐centered cubic (FCC) oxygen framework.^[^ ^5^ ^]^ Accordingly, the (−11‐1) plane of spinel corresponds to the (0001) plane of the original layered structure. Importantly, corundum Co_2_O_3_ coexists with the spinel phase (Figure 4b). In particular, two variants of the corundum phase, that is, [2‐1‐10] and [11‐20], are observed in mirror‐symmetric form. The symmetry originates from the hexagonal system of the corundum phase. The crystallographic orientational relationship among coexisting phases is highlighted in the fast Fourier‐transform (FFT) pattern (Figure 4c). The relationships determined in the FFT pattern are as follows: [121]_spinel_//[2‐1‐10]_corundum_ or [11‐20]_corundum_, and (−11‐1)_spinel_//(0001)_corundum_. Moreover, superimposed [2‐1‐10] and [11‐20] Co_2_O_3_ variants are observed. Figure 4d,e is magnified images of Co_2_O_3_ [2‐1‐10] and the overlapped structure, respectively. Figure 4f shows atomic models of the aforementioned phases. In addition, the FFT pattern matches diffraction pattern *w*_2_ in Figure 3d. Figure S14, Supporting Information, supports the crystallographic orientational relationship. It shows the diffraction pattern of the overcharged LCO viewed along the [11‐20] direction of the R‐3m LCO, which is rotated 30° from the [10‐10] direction; at the same time, Co_2_O_3_ is observed in the [10‐10]_corundum_ direction, which is identical to the above orientational relationship.

The formation of corundum and spinel cobalt oxides is understood based on their structural similarity. Spinel Co_3_O_4_ is a common byproduct of cobalt‐ion migration into lithium‐ion sites during operation.^[^ ^12^, ^30^, ^31^ ^]^ Therefore, it is not surprising that the spinel phase and R‐3m layered phase share a plane with similar structure, although they differ in terms of space group. As mentioned above, these two structures share an FCC oxygen framework. The oxygen framework is a key component for classifying oxides. For example, the several different forms of aluminum oxide can be classified into hexagonal close‐packed (HCP) and FCC oxygen frameworks.^[^ ^32^ ^]^ The most stable form (*α*‐Al_2_O_3_) has a corundum structure with an HCP oxygen framework. On the other hand, the trivalent cobalt oxide Co_2_O_3_ of the same corundum structure has been reported to be unstable; thus, neither the phase itself nor its formation has been studied as intensively as the spinel phase.^[^ ^33^, ^34^ ^]^ The transformation of cobalt oxides was elucidated by comparison with the corundum *α*‐Al_2_O_3_ and spinel *γ*‐Al_2_O_3_ transformation; the crystallographic orientational relationship in the case of alumina is consistent with our result.^[^ ^32^, ^35^ ^]^ Thus, in the case of cobalt oxide, the formation of Co_3_O_4_ first occurs via cation migration without changes in oxygen stacking. Due to the high oxidation potential during overcharging, trivalent Co(III)_2_O_3_ with a higher oxidation state (+3) becomes more favorable than the spinel phase (+2.67) even though they differ in oxygen stacking. Therefore, the transformation from spinel to corundum is accomplished via shifting the oxygen framework, in which two symmetric Co_2_O_3_ variants are equally favorable as observed herein.

**Figure 5:**
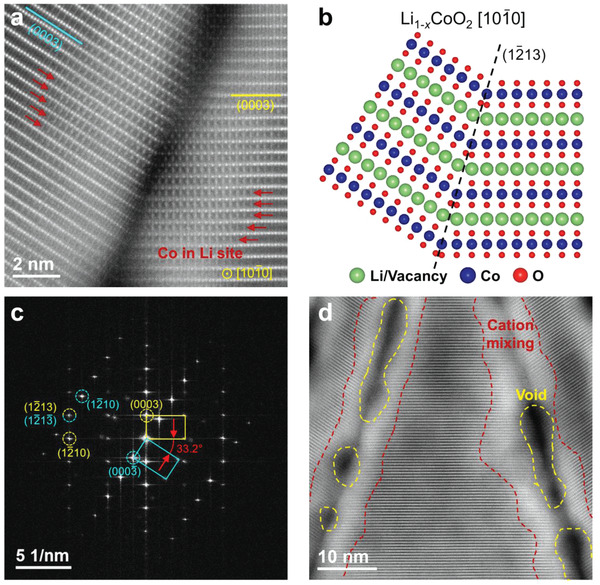
Twin‐like grain boundary of the wedge‐shaped crack. a) Enlarged high‐angle annular dark‐field scanning transmission electron microscopy (HAADF‐STEM) image of the crack boundary. The (0003) plane of each grain is marked in different colors. Cobalt ions in the lithium ion site are indicated by red arrows. b) Atomic‐model structure of the twin boundary with the twin plane of (1‐213) based on the [10‐10] R‐3m structure. c) Fast Fourier‐transformation pattern of (a) showing overlap of the {1‐213} peaks. The same color in (a) is used to distinguish different grains. The misorientation angle is 33.2°. d) HAADF‐STEM image showing cation mixing near the crack boundary. The cation mixing region shows blurred contrast, which is highlighted by the red‐dotted area. The yellow‐dotted area indicates a void‐type defect and appears with dark contrast.

The wedge‐shaped crack, and particularly its twin‐like grain boundary, was investigated in detail. The darker region, where the crack was located, took the form of the layered R‐3m structure, as mentioned earlier. **Figure** **5** reveals that the grain boundary was formed by deformation of the (0003) layer; in particular, it displays a twin‐like boundary. Figure 5a shows an atomic‐resolution HAADF‐STEM image of the grain boundary. Figure 5b displays the corresponding atomic model of the twin boundary based on the R‐3m structure. It shows that the two [10‐10] variants share a common plane (1‐213) as a twin plane, confirmed by the FFT pattern in Figure 5c, which also agrees with the NMF result shown in Figure 3d. Here, the measured angle of misorientation is 33.2°, which is consistent with the expected angle of 33.4° according to the ideal twin‐boundary model. However, the grain boundaries are not as stable as bulk area. To confirm the stability of the twin‐like grain boundary, first‐principles calculations were conducted to compare interface energy. The most stable atomic models of the grain boundary are shown in Figure S15a, Supporting Information. Here, the interface energy of [10‐10](1‐213) grain boundary is 0.46 J m^−2^, whereas the interface energy of [11‐20](−1104) grain boundary is 0.3 J m^−2^ which is previously reported as the most common twin boundary.^[^ ^36^ ^]^ The reason why [10‐10](1‐213) grain boundary is formed in place of the other grain boundary despite the higher energy is speculated based on their geometrical discrepancy. It is worth noting the fact that the plane (−1104) and (1‐104) are asymmetric in the case of [11‐20](−1104) grain boundary, while the [10‐10] system has symmetrical nature, as shown in Figure 5d. (Both planes (1‐213) and (−12‐13) act as the twin boundary symmetrically) Thus, [11‐20](−1104) grain boundary is unable to form the wedge‐shaped crack, geometrically.

Cation mixing, that is, cobalt‐ion migration into lithium‐ion sites, is observed in the vicinity of the boundary (Figure 5a). It is known that the cation‐mixing region blocks lithium‐ion transfer,^[^ ^12^, ^37^ ^]^ which leads to the capture of residual lithium inside and results in a local difference in the charge state. Furthermore, cation mixing widely occurred along the grain boundary (Figure 5d). The cation mixing region appears as a blurry region in the mass–thickness contrast part of the HAADF‐STEM image. Moreover, there are numerous void‐type defects (appearing as areas of dark contrast in Figure 5d) at the boundary. The vacancy formation energy of cobalt and lithium is lower at the grain boundaries compared to bulk (Figure S16, Supporting Information). This supports the observation of both cation‐mixing and void, as the cations at the grain boundaries can be extracted and migrate better. Thus, voids intensify the degradation, including cation mixing, because it acts as an electrolyte permeation path. Although it was previously believed that degradation mainly takes place on the surface of the particle, crack formation directly exposes the interior to degradation, emphasizing the importance to prevent it.

**Figure 6:**
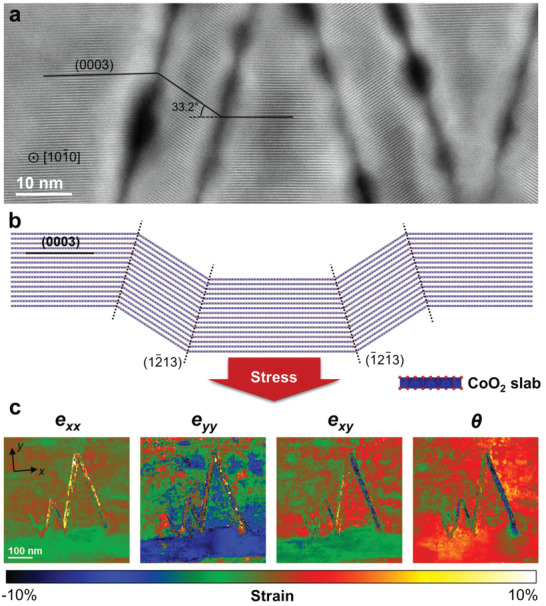
Microstructure of the wedge‐shaped crack. a) High‐angle annular dark‐field scanning transmission electron microscopy (HAADF‐STEM) image showing deformation of the (0003) layer. b) Schematic illustration of the “twin‐like deformation” originating from stress relaxation. c) Strain mapping result of the wedge‐shaped crack acquired by 4D STEM. The *x* and *y* directions are marked in the strain map.

The origin of the wedge‐shaped crack can be further understood by considering stress relaxation phenomena. An HAADF‐STEM image of the crack, highlighting the deformation of the (0003) layer, is presented in **Figure** **6**a. Figure 6b is an atomic‐model structure of the crack based on the CoO_2_ slab. To identify the spatial distribution of the lattice deformation, strain‐mapping analysis of the 4D STEM data was carried out (Figure 6c). For each diffraction pattern of the 4D data set, the variance of two diffraction spots, that is, (−12‐10) and (0006), was measured and compared with the layered R‐3m region as a reference (these two reciprocal vectors formed the *x* and *y* axes of the strain map). These diffraction spots correspond to (−22‐2) and (40‐4) of the Co_3_O_4_ structure, and (0006) and (03‐30) of the Co_2_O_3_ structure, since they share a similar crystal structure. Notably, Co_2_O_3_ shows a remarkable lattice contraction of the (0006) spot. The interlayer spacing of (0003) in Co_2_O_3_ is 4.320 Å, whereas that of LiCoO_2_ and Co_3_O_4_ is 4.685 and 4.667 Å, respectively. This is clearly visible in the *e_yy_* map in Figure 6c. The blue contrast region, where the cobalt oxides are observed as mentioned above, indicates a negative strain of ≈7%, consistent with the prediction (7.8%). The 4D STEM analysis result for the other crack is shown in Figure S17, Supporting Information; the similar strain mapping characteristics support crack formation via stress relaxation. Therefore, we conclude that the wedge‐shaped crack serves to relax stress resulting from cobalt oxide (mainly corundum Co_2_O_3_) formation; moreover, the collapsed cobalt oxide phase leads to permanent capacity fade, since those phases are incapable of lithium insertion.

## 3 Conclusion

The wedge‐shaped crack observed in overcharged LCO proved to be a twin‐like (0003) layer deformation of Li_1−_ *_x_* CoO_2_. Both the shape and deformation mechanism of the crack are reported here for the first time. The abnormal crack was observed at the boundary of separated phases, namely Li_1−_ *_x_* CoO_2_ and cobalt oxides. Here, phase inhomogeneity provoked crack formation. The formation of cobalt oxides, in particular the uncommon corundum structure Co_2_O_3_, caused significant lattice mismatch in *d*_0003_. Accordingly, we suggest that crack formation was due to stress relaxation. Furthermore, overcharged LCO showed evidence of degradation after crack formation, that is, numerous voids in the crack boundary that acted as a path for electrolyte permeation, cation‐mixing that occurred near the voids (which restricted lithium‐ion diffusion), and physical damage to particles that included abnormal cracking vertical to the (0003) plane. Therefore, crack‐proof layered lithium transition‐metal oxide is needed, since a crack can lead to serious irreversible degradation. Moreover, given the current requirement for fast charging, prevention of inhomogeneous charging reactions cannot be overemphasized.

## 4 Experimental Section

### Sample Preparation and Electrochemical Testing

Commercial LCO materials (Sigma‐Aldrich) were used in this study without further treatment. The cathode was prepared by mixing 80 wt% LCO, 10 wt% carbon black, and 10 wt% polyvinylidene fluoride with a sufficient amount of *n*‐methyl‐2‐pyrrolidone solvent. The mixture was coated on aluminum foil, dried for 24 h in a 70 °C vacuum oven, and roll‐pressed. The active material loading mass was 2.69 mg cm^−2^. The CR2032 type coin cells were assembled with LCO cathode, lithium metal foil anode, and glass microfiber filter (Whatman) separator in the glove box. A solution of 1 mol L^−1^ lithium hexafluorophosphate (LiPF_6_) in a 1:1 (in volume) mixture of ethylene carbonate and dimethyl carbonate was used as the electrolyte. Electrochemical measurements were performed under constant‐current conditions with a specific current of 150 mA g^−1^ (1 C‐rate), by changing the cut‐off voltage. To avoid unexpected variables, electrochemical tests were conducted without precycles.

### TEM Measurements

Cross‐sectional TEM specimens were prepared using a focused ion beam (Helios 650; FEI) and thinned using a low‐energy ion‐milling system (NanoMill M1040; Fischione). Scanning electron nanodiffraction measurements were accomplished used a 200‐kV field‐emission TEM (JEM‐2100F; JEOL) equipped with an ASTAR (NanoMEGAS) device. Atomic‐resolution images were acquired by double spherical‐aberration (Cs)‐corrected STEM (ThemisZ; Thermo Fisher Scientific) operating at 200 kV with convergence semi‐angle of 18 mrad. The collection angle for the HAADF‐STEM ranged from 54 to 200 mrad. The NMF analysis for 4D STEM was carried out in Python using the Scikit‐learn package.^[^ ^38^ ^]^ Strain mapping was achieved using the algorithm provided in the Gatan Microscopy Suite (Gatan).

### First Principles Calculations

First principles calculations were performed using the Vienna ab initio simulation package.^[^ ^39^ ^]^ The projector‐augmented wave method was used to describe electron–ion interactions.^[^ ^40^ ^]^ The generalized gradient approximation was used to treat the exchange‐correlation energy.^[^ ^41^ ^]^ The on‐site energy, *U*_eff_, of 4.91 eV for the Co 3d states was taken following the previous report.^[^ ^36^ ^]^ The cutoff energy for the plane‐wave basis set was set to 520 eV. Spin‐polarized calculations were conducted for all models.

### Structural Characterization

Particle morphology was observed by field‐emission SEM (SU70; Hitachi). Raman spectroscopy was performed using a Raman microscope (LabRAM HR Evolution; HORIBA) with 532 nm radiation. The XRD data were acquired using a Smart Lab diffractometer (Rigaku) equipped with a Mo K*α* source.

## Conflict of Interest

The authors declare no conflict of interest.

## Supporting information

Supporting Information

## Data Availability

The data that support the findings of this study are available from the corresponding author upon reasonable request.

## References

[1] N. Nitta , F. Wu , J. T. Lee , G. Yushin , Mater. Today 2015, 18, 252.

[2] T. Placke , R. Kloepsch , S. Dühnen , M. Winter , J. Solid State Electrochem. 2017, 21, 1939.

[3] K. Mizushima , P. C. Jones , P. J. Wiseman , J. B. Goodenough , Mater. Res. Bull. 1980, 15, 783.

[4] P. He , H. Yu , D. Li , H. Zhou , J. Mater. Chem. 2012, 22, 3680.

[5] M. D. Radin , S. Hy , M. Sina , C. Fang , H. Liu , J. Vinckeviciute , M. Zhang , M. S. Whittingham , Y. S. Meng , A. Van der Ven , Adv. Energy Mater. 2017, 7, 1602888.

[6] Y. Lyu , X. Wu , K. Wang , Z. Feng , T. Cheng , Y. Liu , M. Wang , R. Chen , L. Xu , J. Zhou , Y. Lu , B. Guo , Adv. Energy Mater. 2021, 11, 2000982.

[7] W. Li , B. Song , A. Manthiram , Chem. Soc. Rev. 2017, 46, 3006. 28440379 10.1039/c6cs00875e

[8] K. Liu , Y. Liu , D. Lin , A. Pei , Y. Cui , Sci. Adv. 2018, 4, eaas9820. 29942858 10.1126/sciadv.aas9820 PMC6014713

[9] R. Hausbrand , G. Cherkashinin , H. Ehrenberg , M. Gröting , K. Albe , C. Hess , W. Jaegermann , Mater. Sci. Eng. B 2015, 192, 3.

[10] H. Liu , M. Wolf , K. Karki , Y.‐S. Yu , E. A. Stach , J. Cabana , K. W. Chapman , P. J. Chupas , Nano Lett. 2017, 17, 3452. 28548836 10.1021/acs.nanolett.7b00379

[11] P. Yan , J. Zheng , M. Gu , J. Xiao , J.‐G. Zhang , C.‐M. Wang , Nat. Commun. 2017, 8, 14101. 28091602 10.1038/ncomms14101 PMC5241805

[12] A. Yano , M. Shikano , A. Ueda , H. Sakaebe , Z. Ogumi , J. Electrochem. Soc. 2017, 164, A6116.

[13] H.‐H. Ryu , K.‐J. Park , C. S. Yoon , Y.‐K. Sun , Chem. Mater. 2018, 30, 1155.

[14] D. P. Finegan , M. Scheel , J. B. Robinson , B. Tjaden , M. Di Michiel , G. Hinds , D. J. L. Brett , P. R. Shearing , Phys. Chem. Chem. Phys. 2016, 18, 30912. 27388638 10.1039/c6cp04251a

[15] D. Ren , X. Feng , L. Lu , M. Ouyang , S. Zheng , J. Li , X. He , J. Power Sources 2017, 364, 328.

[16] Y. Xu , E. Hu , K. Zhang , X. Wang , V. Borzenets , Z. Sun , P. Pianetta , X. Yu , Y. Liu , X.‐Q. Yang , H. Li , ACS Energy Lett. 2017, 2, 1240.

[17] Y. Zhang , Z. Yang , C. Tian , J. Mater. Chem. A 2019, 7, 23628.

[18] X. Dai , A. Zhou , J. Xu , Y. Lu , L. Wang , C. Fan , J. Li , J. Phys. Chem. C 2016, 120, 422.

[19] A. Liu , J. Li , R. Shunmugasundaram , J. R. Dahn , J. Electrochem. Soc. 2017, 164, A1655.

[20] Q. Liu , X. Su , D. Lei , Y. Qin , J. Wen , F. Guo , Y. A. Wu , Y. Rong , R. Kou , X. Xiao , F. Aguesse , J. Bareño , Y. Ren , W. Lu , Y. Li , Nat. Energy 2018, 3, 936.

[21] S. Sharifi‐Asl , F. A. Soto , T. Foroozan , M. Asadi , Y. Yuan , R. Deivanayagam , R. Rojaee , B. Song , X. Bi , K. Amine , J. Lu , A. Salehi‐khojin , P. B. Balbuena , R. Shahbazian‐Yassar , Adv. Funct. Mater. 2019, 29, 1901110.

[22] D. Kramer , G. Ceder , Chem. Mater. 2009, 21, 3799.

[23] Y. Kim , H. Lee , S. Kang , J. Mater. Chem. 2012, 22, 12874.

[24] Y. Park , S. H. Shin , S. M. Lee , S. P. Kim , H. C. Choi , Y. M. Jung , J. Mol. Struct. 2014, 1069, 183.

[25] M. Otoyama , Y. Ito , A. Hayashi , M. Tatsumisago , J. Power Sources 2016, 302, 419.

[26] Z. Wang , H. Dong , L. Chen , Y. Mo , X. Huang , Solid State Ionics 2004, 175, 239.

[27] Y. Takahashi , N. Kijima , K. Tokiwa , T. Watanabe , J. Akimoto , J. Phys.: Condens. Matter 2007, 19, 436202.

[28] D. D. Lee , H. S. Seung , Nature 1999, 401, 788. 10548103 10.1038/44565

[29] F. Uesugi , S. Koshiya , J. Kikkawa , T. Nagai , K. Mitsuishi , K. Kimoto , Ultramicroscopy 2021, 221, 113168. 33290980 10.1016/j.ultramic.2020.113168

[30] J. Kikkawa , S. Terada , A. Gunji , T. Nagai , K. Kurashima , K. Kimoto , J. Phys. Chem. C 2015, 119, 15823.

[31] H. Tan , S. Takeuchi , K. K. Bharathi , I. Takeuchi , L. A. Bendersky , ACS Appl. Mater. Interfaces 2016, 8, 6727. 26911456 10.1021/acsami.5b12025

[32] S. Adachi , M. Ishimaru , Y. Sina , C. J. McHargue , K. E. Sickafus , E. Alves , Nucl. Instrum. Methods Phys. Res. B 2015, 358, 136.

[33] J. Chenavas , J. C. Joubert , M. Marezio , Solid State Commun. 1971, 9, 1057.

[34] V. Singh , D. T. Major , Inorg. Chem. 2016, 55, 3307. 27010797 10.1021/acs.inorgchem.5b02426

[35] P. F. Yan , K. Du , M. L. Sui , Acta Mater. 2010, 58, 3867.

[36] H. Moriwake , A. Kuwabara , C. A. J. Fisher , R. Huang , T. Hitosugi , Y. H. Ikuhara , H. Oki , Y. Ikuhara , Adv. Mater. 2013, 25, 618. 23125001 10.1002/adma.201202805

[37] H. Wang , Y.‐I. Jang , B. Huang , D. R. Sadoway , Y.‐M. Chiang , J. Electrochem. Soc. 1999, 146, 473.

[38] F. Pedregosa , G. Varoquaux , A. Gramfort , V. Michel , B. Thirion , O. Grisel , M. Blondel , P. Prettenhofer , R. Weiss , V. Dubourg , J. Vanderplas , A. Passos , D. Cournapeau , J. Mach. Learn. Res. 2011, 12, 2825.

[39] G. Kresse , J. Furthmüller , Phys. Rev. B 1996, 54, 11169. 10.1103/physrevb.54.11169 9984901

[40] P. E. Blöchl , Phys. Rev. B 1994, 50, 17953. 10.1103/physrevb.50.17953 9976227

[41] J. P. Perdew , K. Burke , M. Ernzerhof , Phys. Rev. Lett. 1996, 77, 3865. 10062328 10.1103/PhysRevLett.77.3865

