# Supplementary material for: Overcharge‐Induced Phase Heterogeneity and Resultant Twin‐Like Layer Deformation in Lithium Cobalt Oxide Cathode for Lithium‐Ion Batteries
Source: Adv Sci (Weinh). 2022 Sep 11;9(32):2203639. doi: 10.1002/advs.202203639 (PMC9661829; doi:10.1002/advs.202203639)
Supplement: Supplementary file 1 — Supporting Information [file ADVS-9-2203639-s001.pdf]

Supporting Information

Overcharge-induced Phase Heterogeneity and Resultant Twin-like Layer Deformation in Lithium Cobalt Oxide Cathode for Lithium-ion Batteries

*Juhyun Oh, Seung-Yong Lee, Hwangsun Kim, Jinseok Ryu, Byeongjun Gil, Jongki Lee, and Miyoung Kim**

Excess capacity is observed during overcharging from 5.70 V to 5.79 Vas shown in Figure 1a, and it is understood by the electrolyte decomposition. Linear sweep voltammetry data (**Figure S1**) supports the electrolyte decomposition. Here, the decomposition begins from 5.7 V (manifests as a steep incline) which is in good agreement with the previous study;[1] it reported that the onset for oxidation of ethylene carbonate (EC) and dimethyl carbonate (DMC), which are the main components of the electrolyte, is 5.4 V (vs. Li) with peak potential at 5.7 V. Therefore, the origin of the extra capacity is attributable to the oxidation reaction of electrolyte due to high voltage.

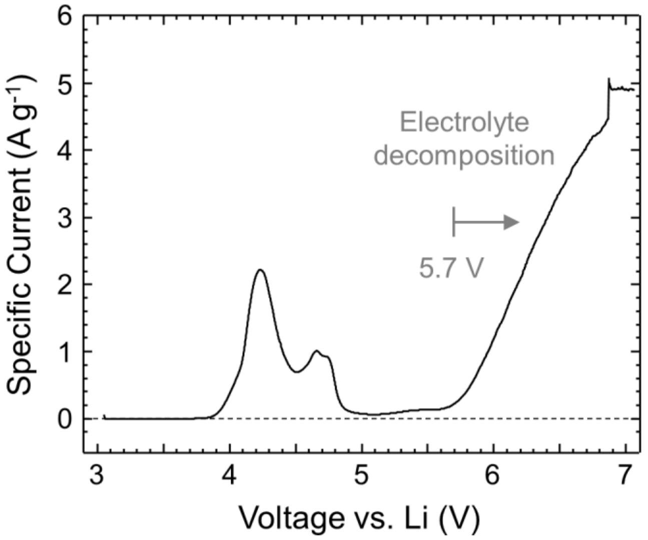

Figure S1. Linear sweep voltammogram of lithium cobalt oxide at a scan rate of 1 mV s^{-1} illustrating electrolyte decomposition above 5.7 V.

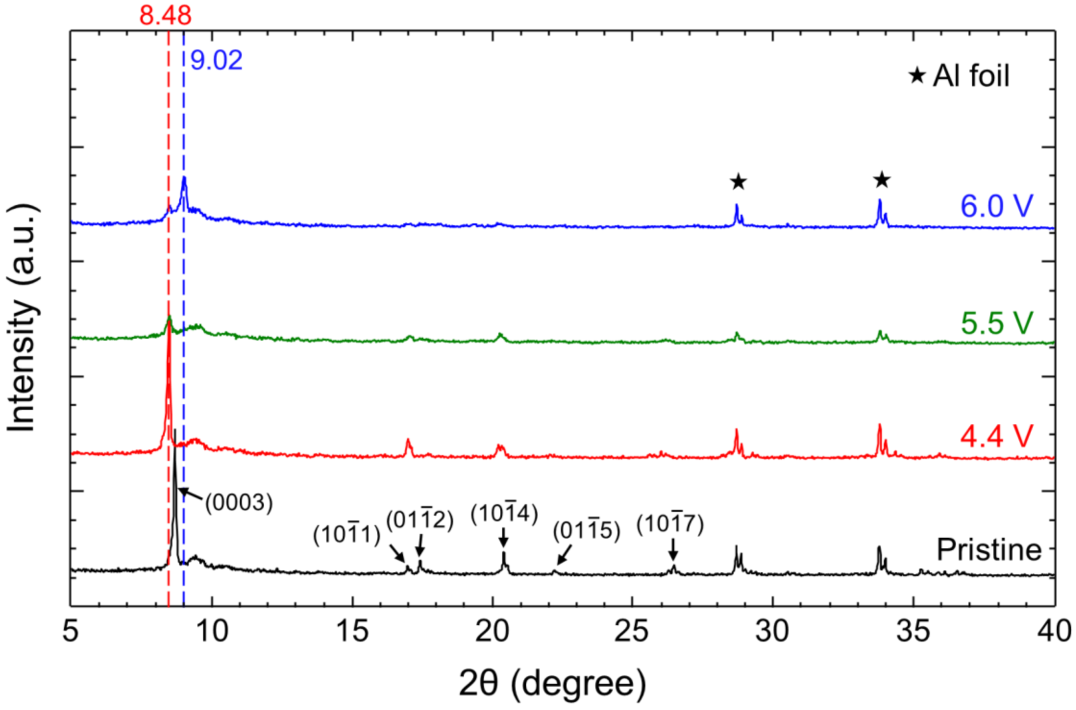

Figure S2. Full X-ray diffraction (XRD) plots of pristine, 4.4-, 5.5-, and 6.0-V cut-off samples. Mo Kα radiation was used.

Additional charging experiments were conducted in the condition consisting of constant current charging of 1 C-rate with a cut-off voltage of 5.5 V and subsequent constant voltage charging of 5.5 V for 10 hours. After charging, the sample (hereafter referred to as the “5.5 V 10 h sample”) has been investigated by scanning electron microscopy (SEM), X-ray diffraction (XRD), Raman spectroscopy, and transmission electron microscopy (TEM) in comparison with the original 5.5 V and 6.0 V results. (**Figure S3-5**) The SEM images in Figure S3 show a similar damage type with the 5.5 V results as shown in Figure 1f, g, i.e. cracks horizontal to the (0003) layer which is indicated here by yellow arrows. In Figure S4a, XRD results are compared with the 5.5 V and 6.0 V samples. The 5.5 V 10 h sample shows a slight peak shift compared to the 6.0 V sample. This implies that the structural change of the 5.5 V 10 h sample is between the level of the 5.5 V sample and the 6.0 V sample. Raman spectra also confirm the tendency as shown in Figure S4b. The Raman spectrum of the 5.5 V 10 h sample matches that of the 5.5 V sample. Furthermore, in the TEM results (Figure S5), there are cracks parallel to the (0003) plane, which has been frequently observed as a result of the degradation.[2,3] Therefore, the measurements of the 5.5 V 10 h sample indicate that the overcharging voltage of 6.0 V is the main cause of the wedge-shaped crack.

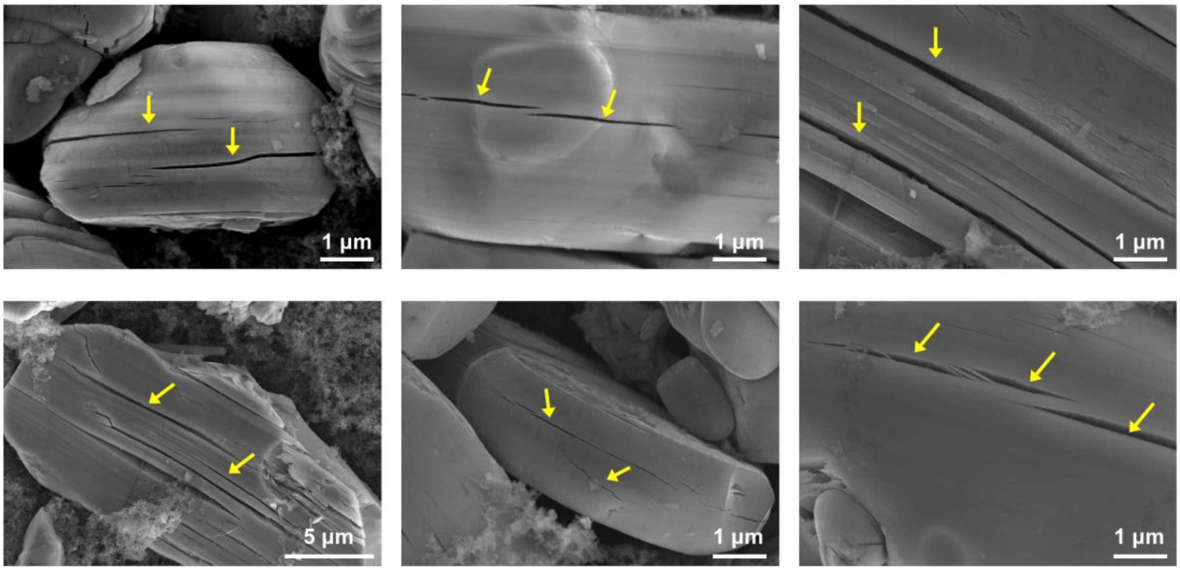

Figure S3. Scanning electron microscopy images of the 5.5 V 10 h sample showing cracks horizontal to the (0003) layer which is indicated by yellow arrows.

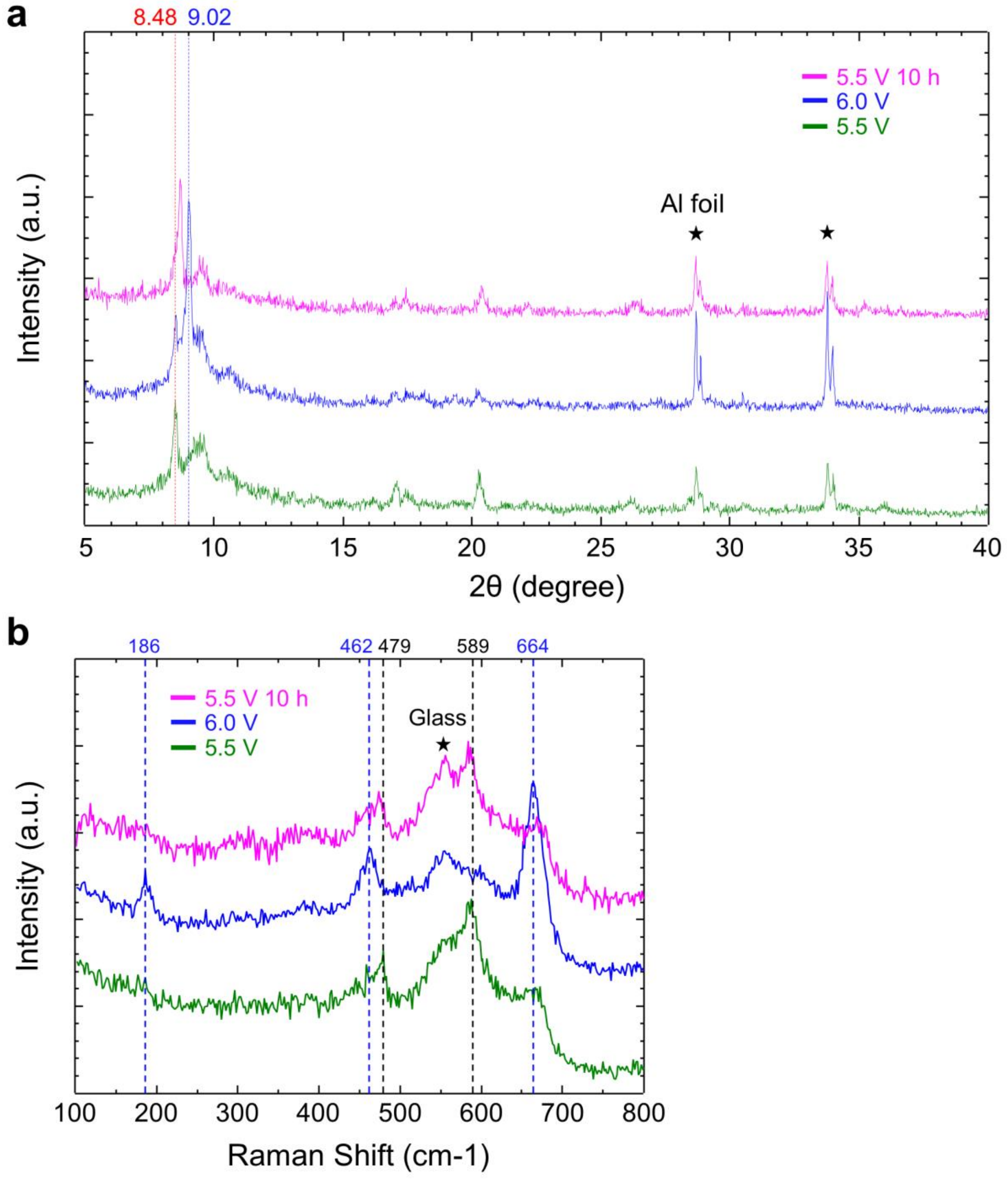

Figure S4. Comparison of lithium cobalt oxide (LCO) among different states of charging. **(a)** XRD plots of 5.5-, 6.0-V cut-off, and 5.5 V 10 h samples showing the (0003) peak variation. Mo Kα radiation was used. **(b)** Raman spectra of 5.5-, 6.0-V cut-off, and 5.5 V 10 h samples. Laser excitation of 532-nm radiation was used. Samples were sealed in glass to prevent air exposure.

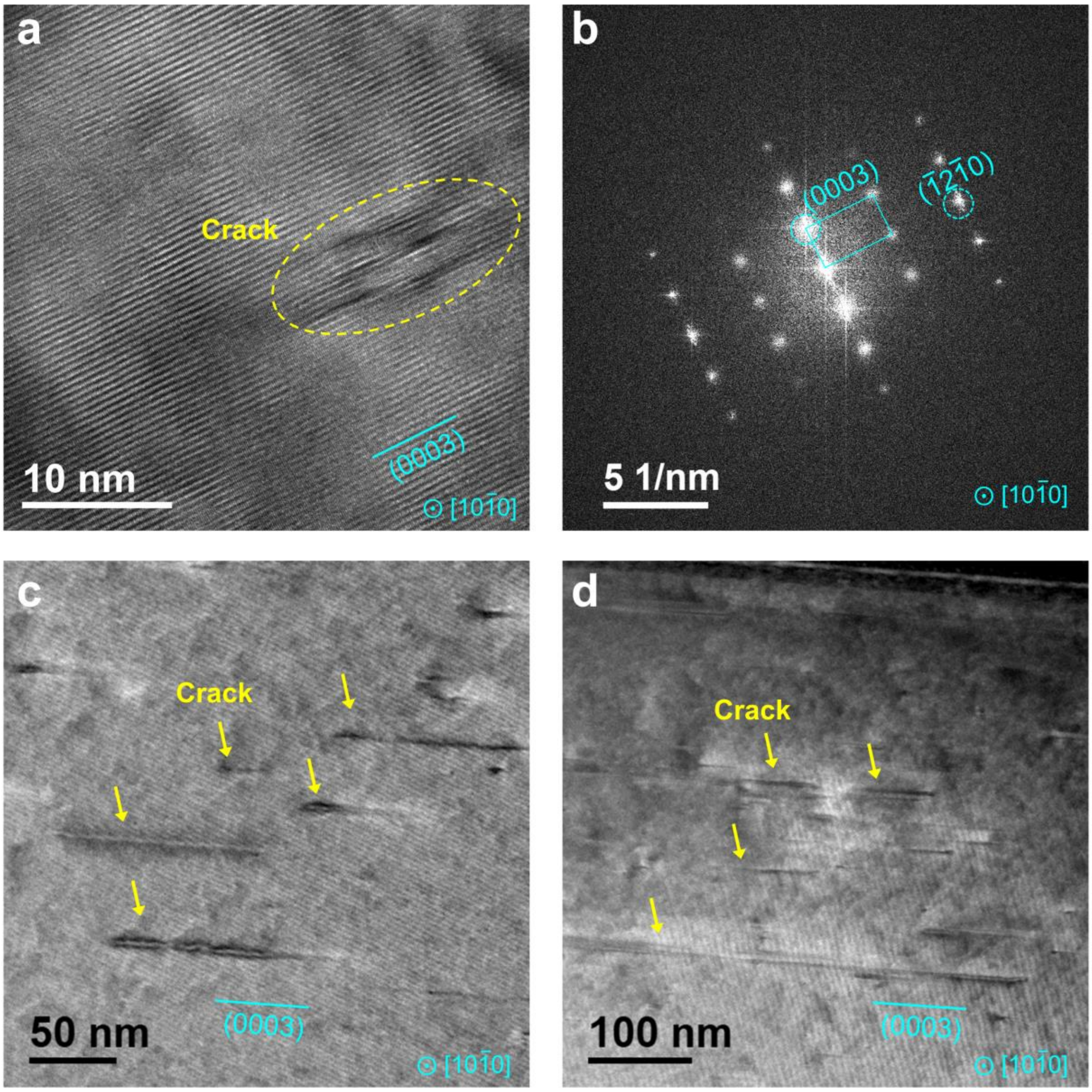

Figure S5. Transmission electron microscopy (TEM) observations of the 5.5 V 10 h sample. **(a)** High-resolution TEM image of the crack parallel to the (0003) plane in the [10-10] grain. **(b)** FFT pattern of (a) confirming [10-10] grain. **(c, d)** High-angle annular dark-field (HAADF) scanning transmission electron microscopy (STEM) images of the cracks parallel to the (0003) plane.

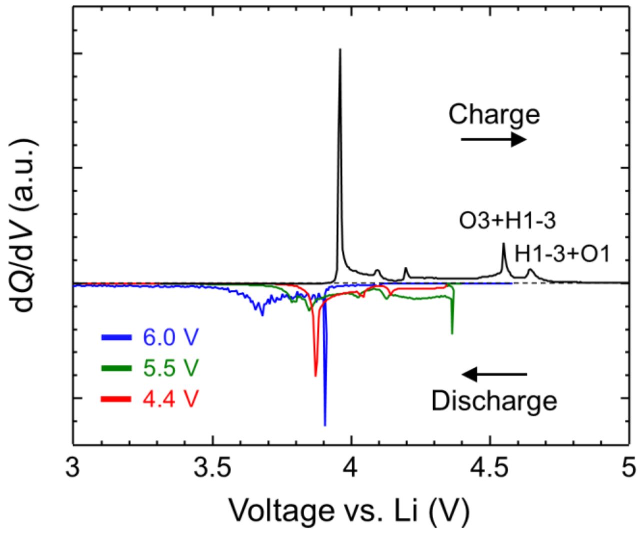

Figure S6. Differential capacity plots (d*Q*/d*V*) of the overcharge and discharge profiles presented in Figures 1a and 2c.

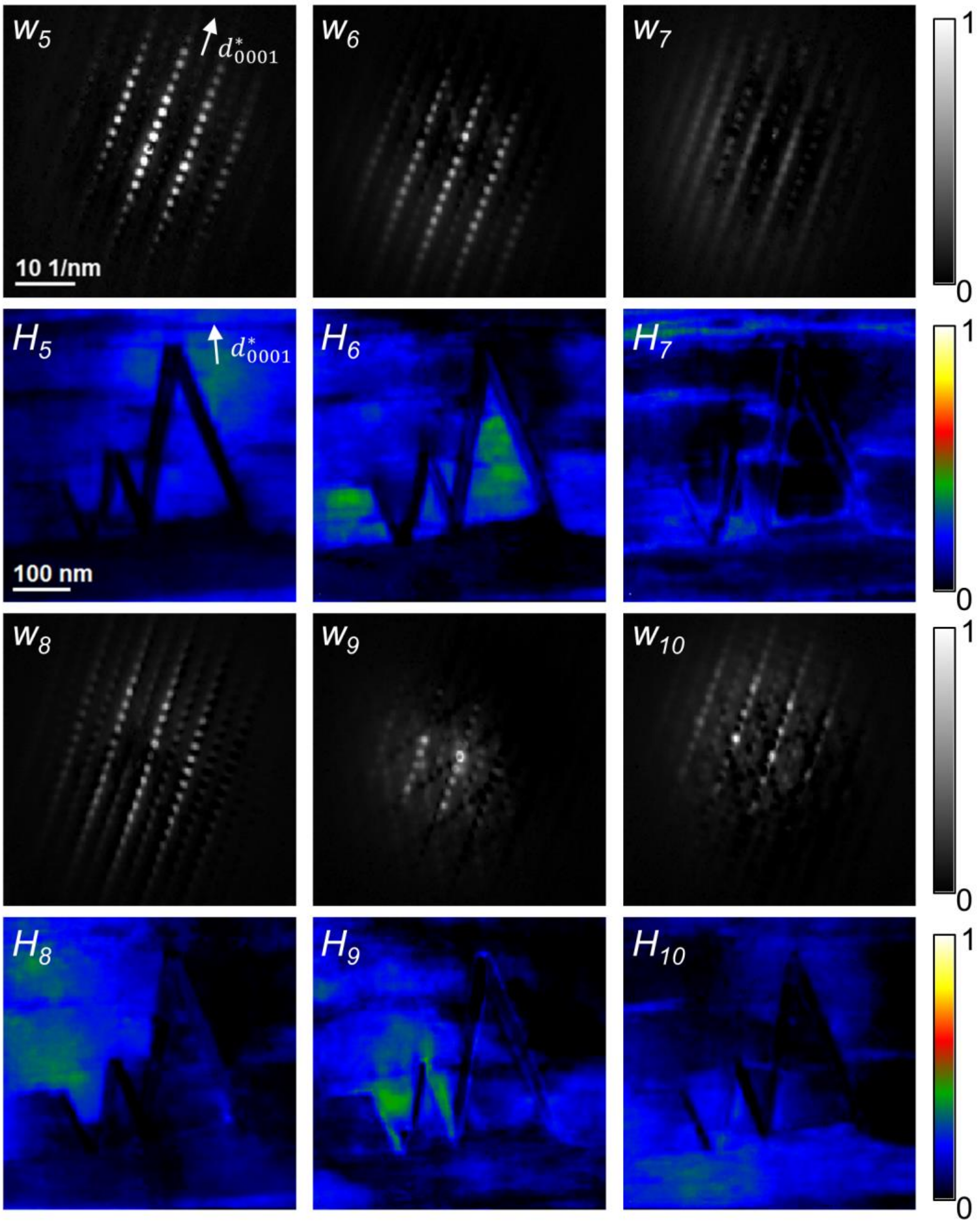

Figure S7. The rest of the non-negative matrix factorization (NMF) results for the 4D STEM data of the wedge-shaped crack. Each of the coefficient maps (H_i) is displayed below its corresponding components (w_i) with normalized intensity. The direction of the reciprocal vector d^*_{0001} is indicated.

The non-negative matrix factorization (NMF) is used to discriminate both phase boundary and grain boundary at the same time in the 4D scanning transmission electron microscopy (STEM) data. It is important to treat such variables (e.g. number of components) properly for successful factorization of the diffraction patterns. As shown in Figure 3d, the condition of ten components is used, and to explain the suitability for this condition, the NMF results are compared by varying the number of components to 5, 10, 15, 20, and 25. **Figure S8** shows the mean square error change during the converging in different numbers of components, *k*. Increasing the number of components drops the error but at the same time, not much decrease is observed after reaching *k*=10. Full NMF data for each condition of *k*=5, 10, and 15 are compared to examine the difference and how the factorization is successful. (**Figure S9, S10**) Comparing Figure S9 and Figure 3d, **S7**, one can find that the third component in Figure S9 (w_3) corresponds to the combination of two components (w_3, w_4) in Figure 3d. Hence, an insufficient number of components leads to erroneous interpretation of the data. On the other hand, Figure S10 shows no significant changes to the condition of the ten components. The first ten components of Figure S10 match that of Figure 3d, S7, and furthermore the rest does not provide additional information. Therefore, the condition of *k*=10 is used in this study, and components related to phase boundary and grain boundary, i.e. w_1-w_4, are selected among others to reveal the phase heterogeneity and the microstructure of the crack.

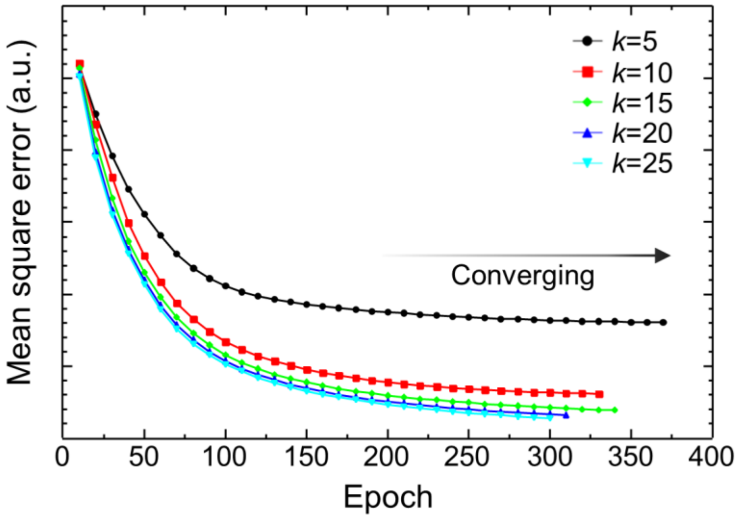

Figure S8. Mean square error as a function of the epoch showing the converging process of NMF. Different condition of the number of components is compared.

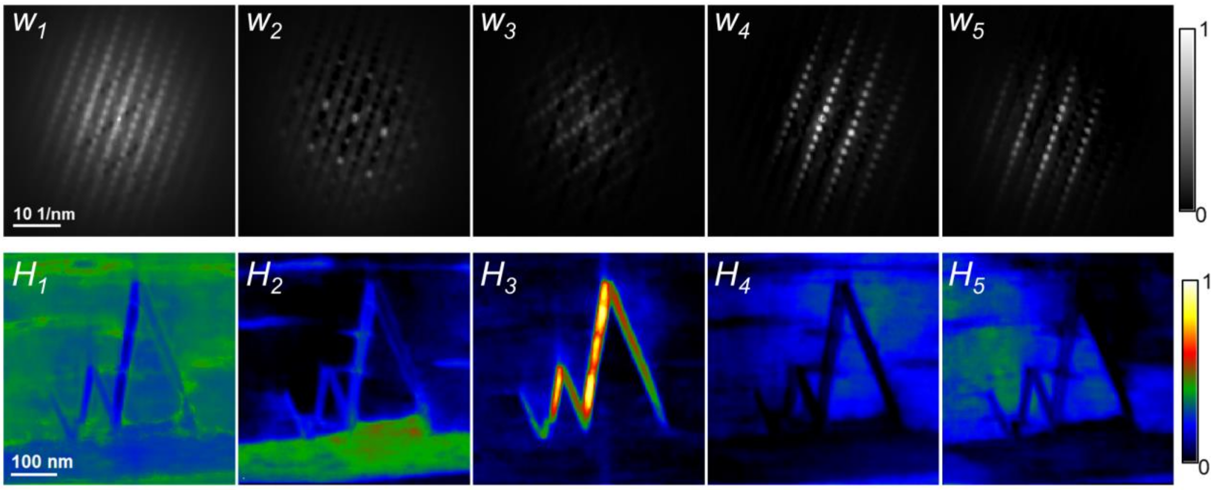

Figure S9. The full NMF results using five components condition. Components (upper row, w_i) and their corresponding coefficient maps (lower row, H_i) are displayed with normalized intensity.

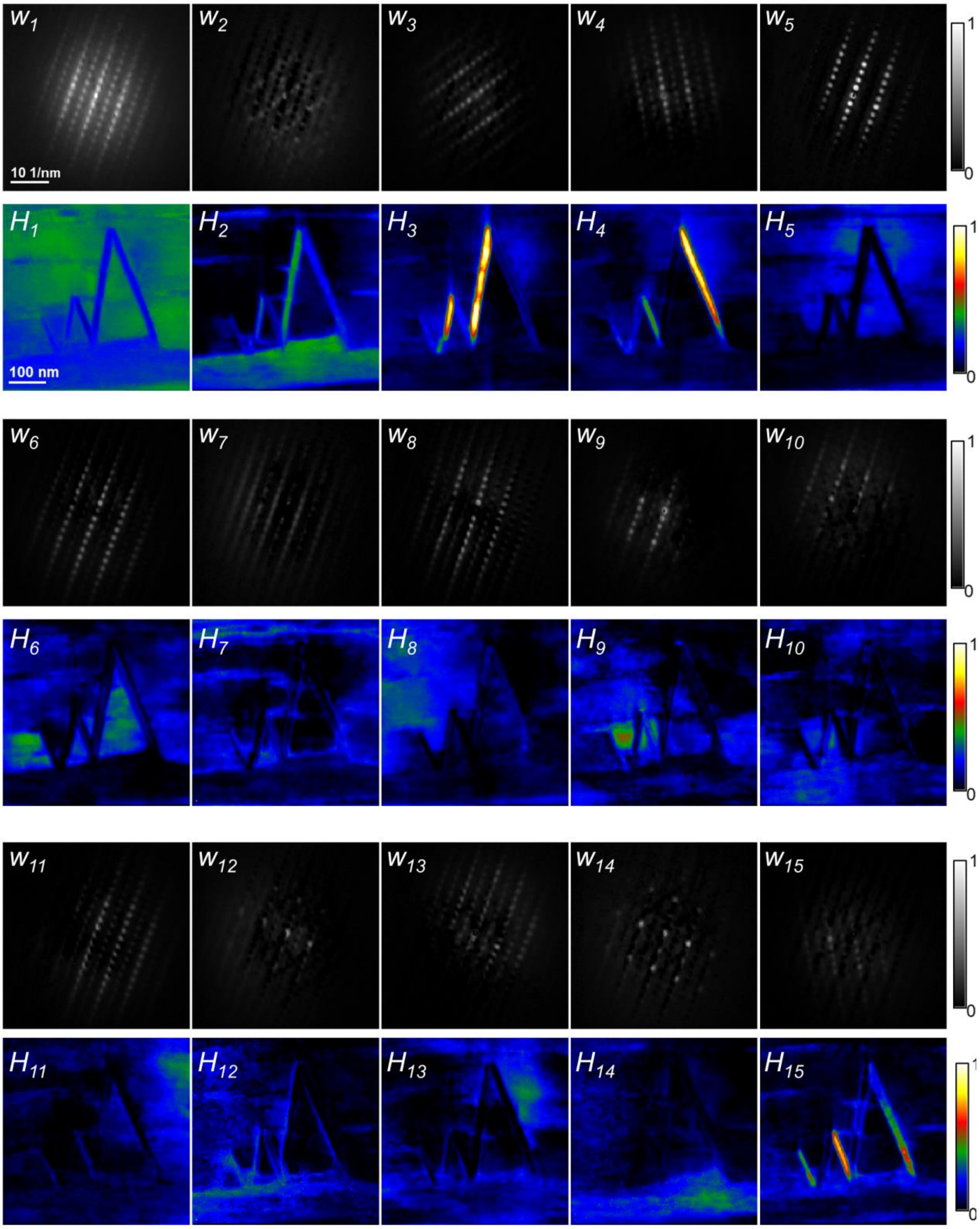

Figure S10. The full NMF results using fifteen components condition. Components (upper row, w_i) and their corresponding coefficient maps (lower row, H_i) are displayed with normalized intensity.

Electron energy loss spectroscopy (EELS) analysis of the area in Figure 3a further revealed heterogeneity in terms of residual lithium content. The EELS spectra in **Figure S11** indicate that there is an evident lithium signal in the darker phase. Following the method of integrating the Li-K edge over the Co-$M_{2,3}$ edge,[4] darker phase regions 1 and 2 show Li-K signals that correspond to Li/Co ratios of 0.215 and 0.135, respectively. On the other hand, the brighter phase (region 3) reveals little Li-K signal and has a Li/Co ratio of 0.020, implying the existence of cobalt oxide phases.

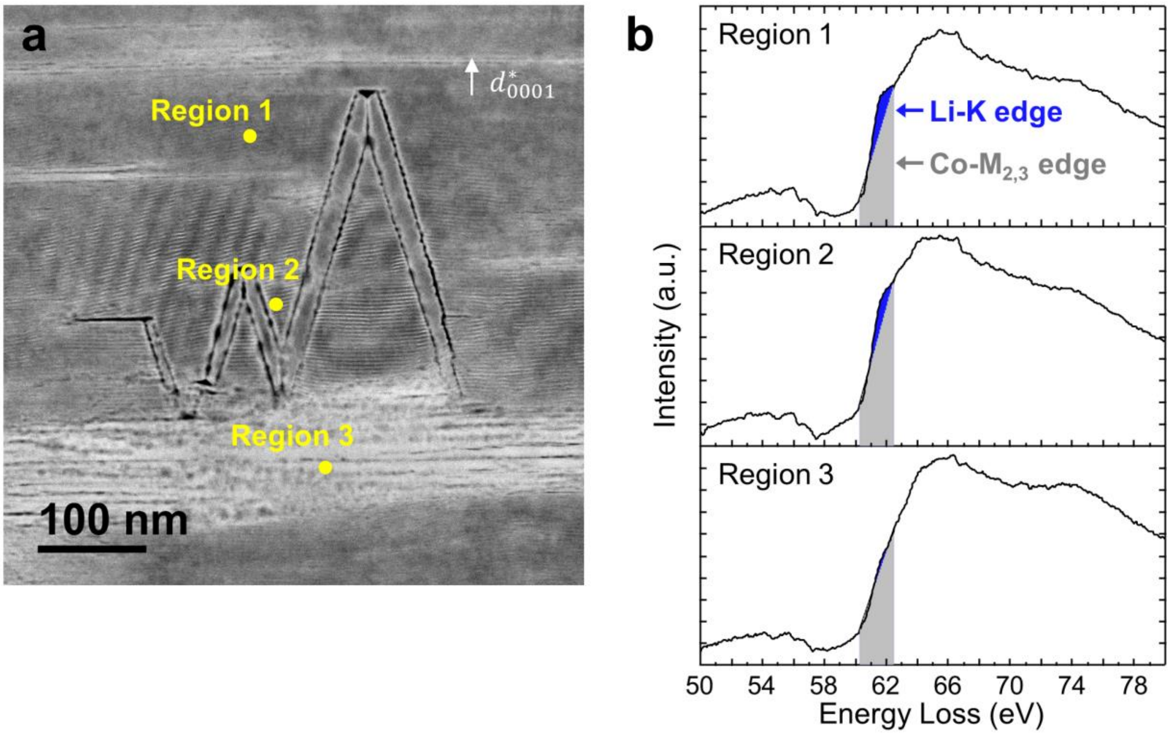

Figure S11. TEM electron energy loss spectroscopy (TEM-EELS) result of the wedge-shaped crack. **(a)** HAADF-STEM image of the wedge-shaped crack. **(b)** EELS spectra of the Co-$M_{2,3}$ edge and Li-K edge acquired from regions 1, 2, and 3 in (a). The direction of the reciprocal vector d^*_{0001} is indicated.

Phase	$LiCoO_2$ R-3m	$Li_{0.5}CoO_2$ P2/m	Co_3O_4 Fd-3m	Co_2O_3 R-3c
Lithium atomic density (*Z*=3) [nm^{-3}]	31.1	15.3	0	0
Cobalt atomic density (*Z*=27) [nm^{-3}]	31.1	30.5	45.5	46.7
Oxygen atomic density (*Z*=8) [nm^{-3}]	62.2	61.1	60.6	70.0

Table S1. Theoretical atomic density of different lithium cobalt oxide phases according to elemental composition.

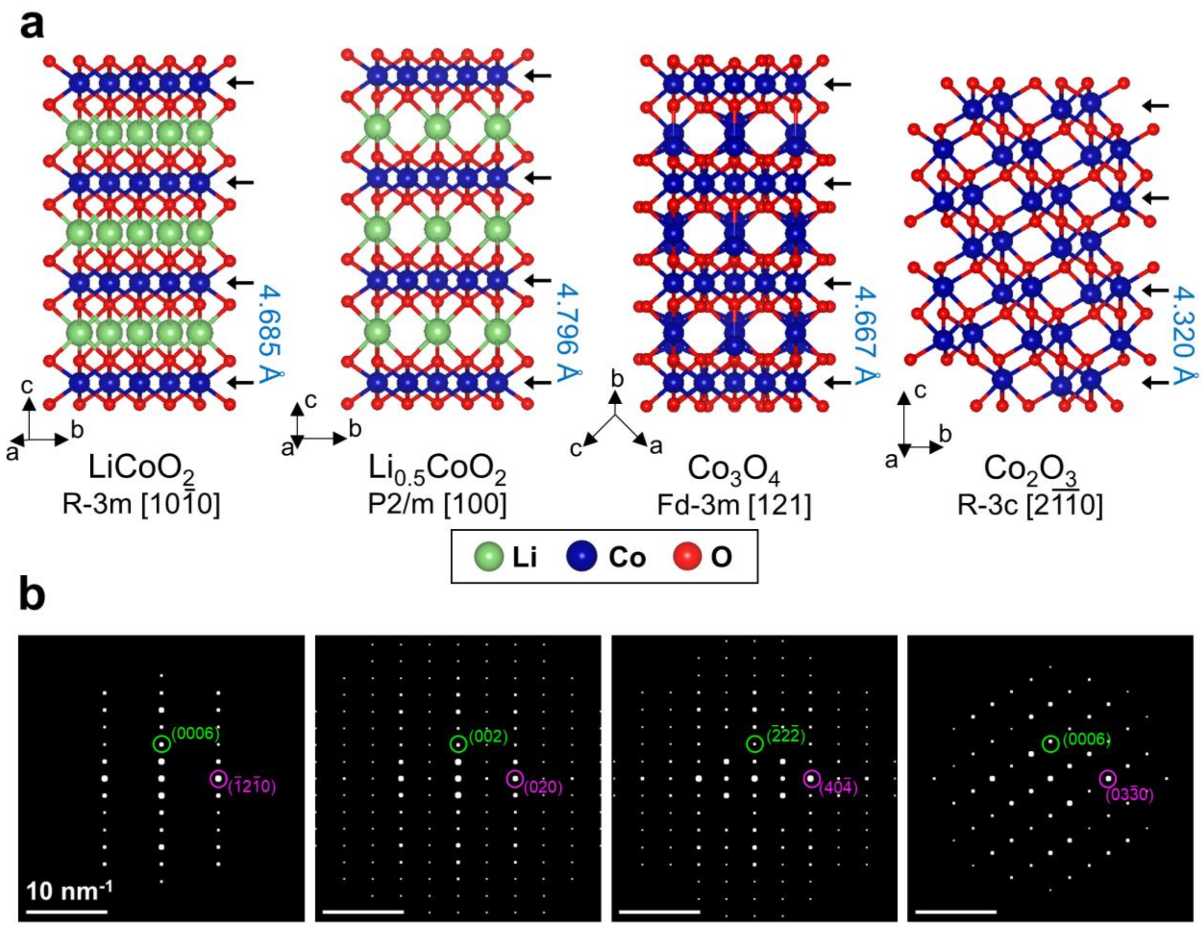

Figure S12. Different phases of lithium cobalt oxide discussed in this study. **(a)** Atomic models of each phase overlaid at the same scale. The black arrows indicate the spacing of the (0003) or (0003)-equivalent slab of each phase. **(b)** The simulated electron diffraction patterns of each phase are displayed below its structure. The green and magenta, circles show the similarity of the phases. The scale bar for the diffraction patterns is 10 nm^{-1}.

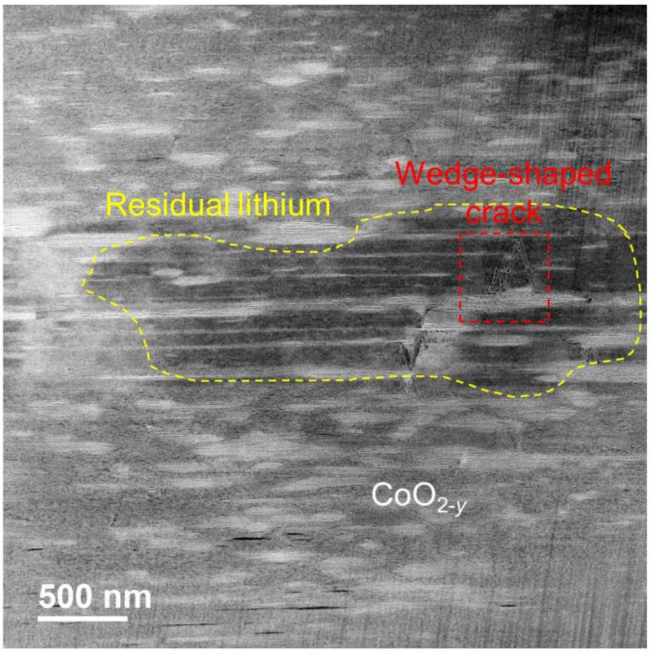

Figure S13. Lower magnification HAADF-STEM image showing that the residual lithium region is enclosed by cobalt oxides. The region indicated by red box corresponds to Figure 3a.

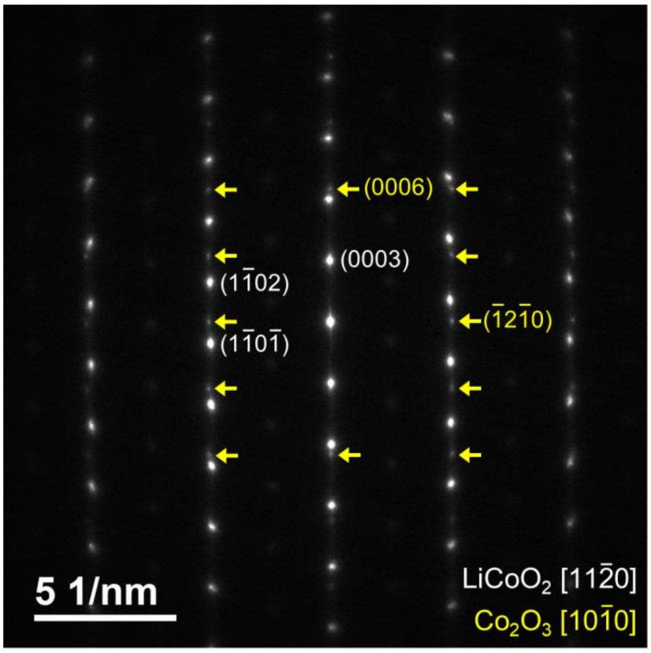

Figure S14. Electron diffraction pattern viewed from a different zone axis showing the crystallographic orientational relationship between Co_2O_3 and $LiCoO_2$.

To identify the twin-like grain boundary clearly, first principles calculations were performed for grain boundary systems, [10-10](1-213) as well as [10-10](1-216), observed in this study. **Figure S15** shows first principles calculations result, i.e. most energetically favorable atomic model structure, and its corresponding grain boundary image observed by high-angle annular dark-field (HAADF) scanning transmission electron microscopy (STEM). Among the possible configurations, the interface energies of grain boundary were compared according to the equation:

$$E_{gb} = \frac{1}{2A}\{E_{supercell} - E_{bulk}\}$$

where A is the surface area of the grain boundary in the supercell composed of two variants, and the factor 2 is to account for two grain boundaries in the supercell. The optimized grain boundary energies of [10-10](1-213) and [10-10](1-216) are 0.46 J m^{-2} and 1.27 J m^{-2}, respectively. Consequently, [10-10](1-216) grain boundary, of which energy is nearly triple of the former, is less observed and shows a more defective interface as shown in Figure S15d.

The vacancy formation energy for each atom (i.e. lithium, cobalt, and oxygen) is calculated at the bulk and grain boundary systems. (**Figure S16**) The vacancy formation energy of lithium and cobalt is decreased in the grain boundary systems compared to bulk. This highlights the unstable nature of grain boundary structure and implies that such side reactions, e.g. defect or cation-mixing, may readily occur. However, oxygen vacancy formation energy shows a modest increase. This suggests that oxygen atoms prefer to promote additional dissociation rather than forming a single vacancy.

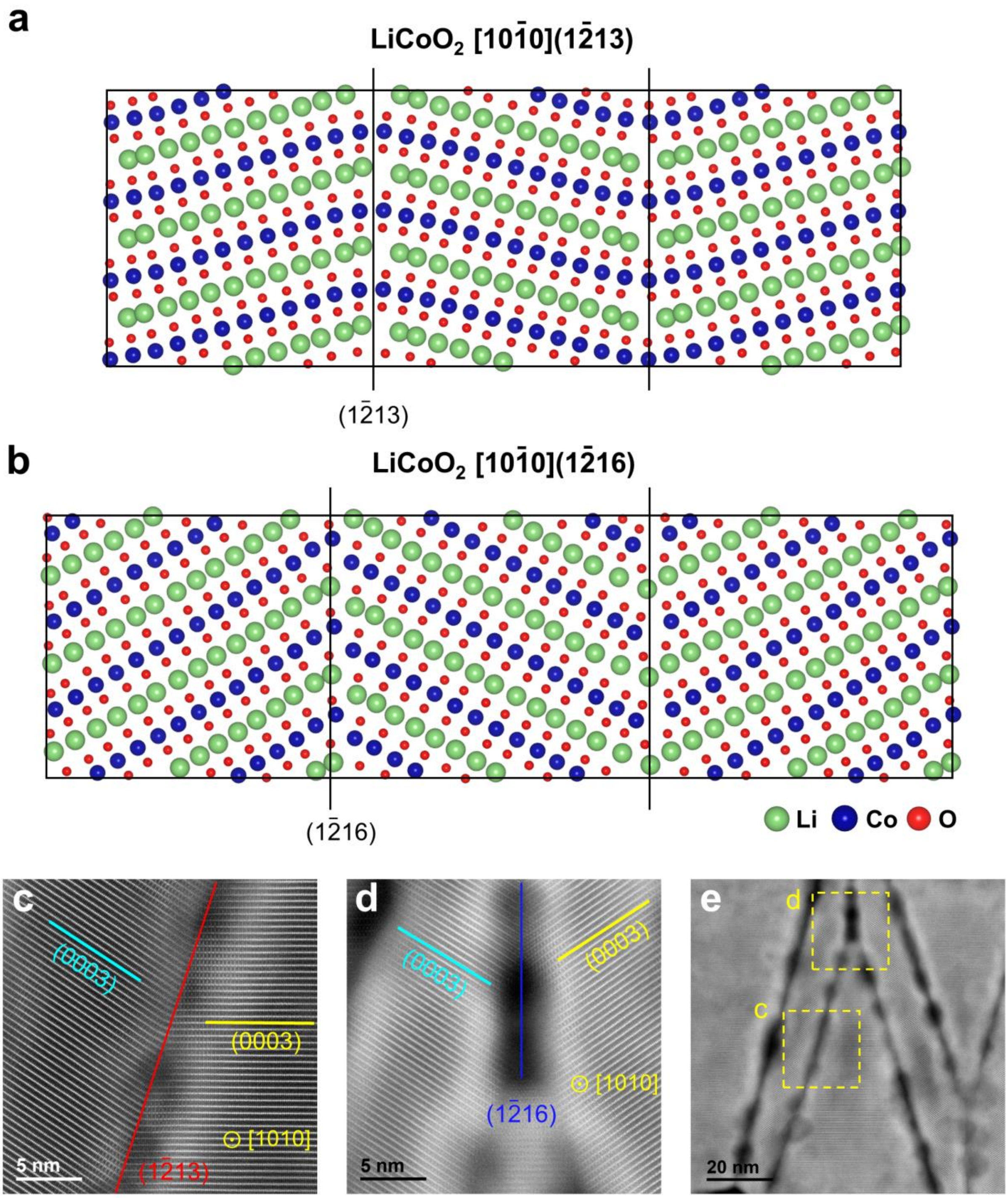

Figure S15. First principles simulations of the atomic model structure of the grain boundary. The most stable atomic configuration of **(a)** [10-10](1-213) twin-like grain boundary with a misorientation angle of 33.4° and **(b)** [10-10](1-216) twin-like grain boundary with a misorientation angle of 62.0°. HAADF-STEM image of **(c)** [10-10](1-213) grain boundary and **(d)** [10-10](1-216) grain boundary in the wedge-shaped crack. **(e)** Lower magnification image showing the part represented in (c) and (d). The interface energy of grain boundary in (a) and (b) is 0.46 J m^{-2} and 1.27 J m^{-2}, respectively.

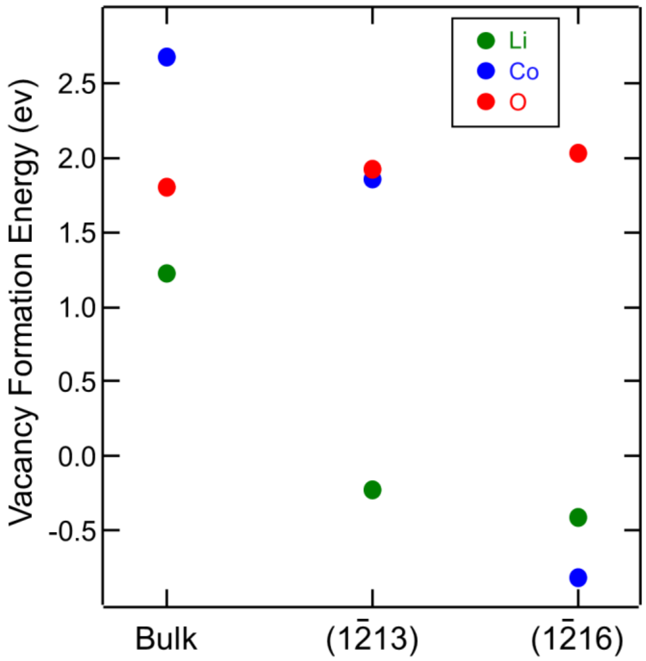

Figure S16. First principles simulations of the vacancy formation energy at the bulk and grain boundary models of [10-10](1-213) and [10-10](1-216). The vacancy formation energy is calculated for each atom respectively.

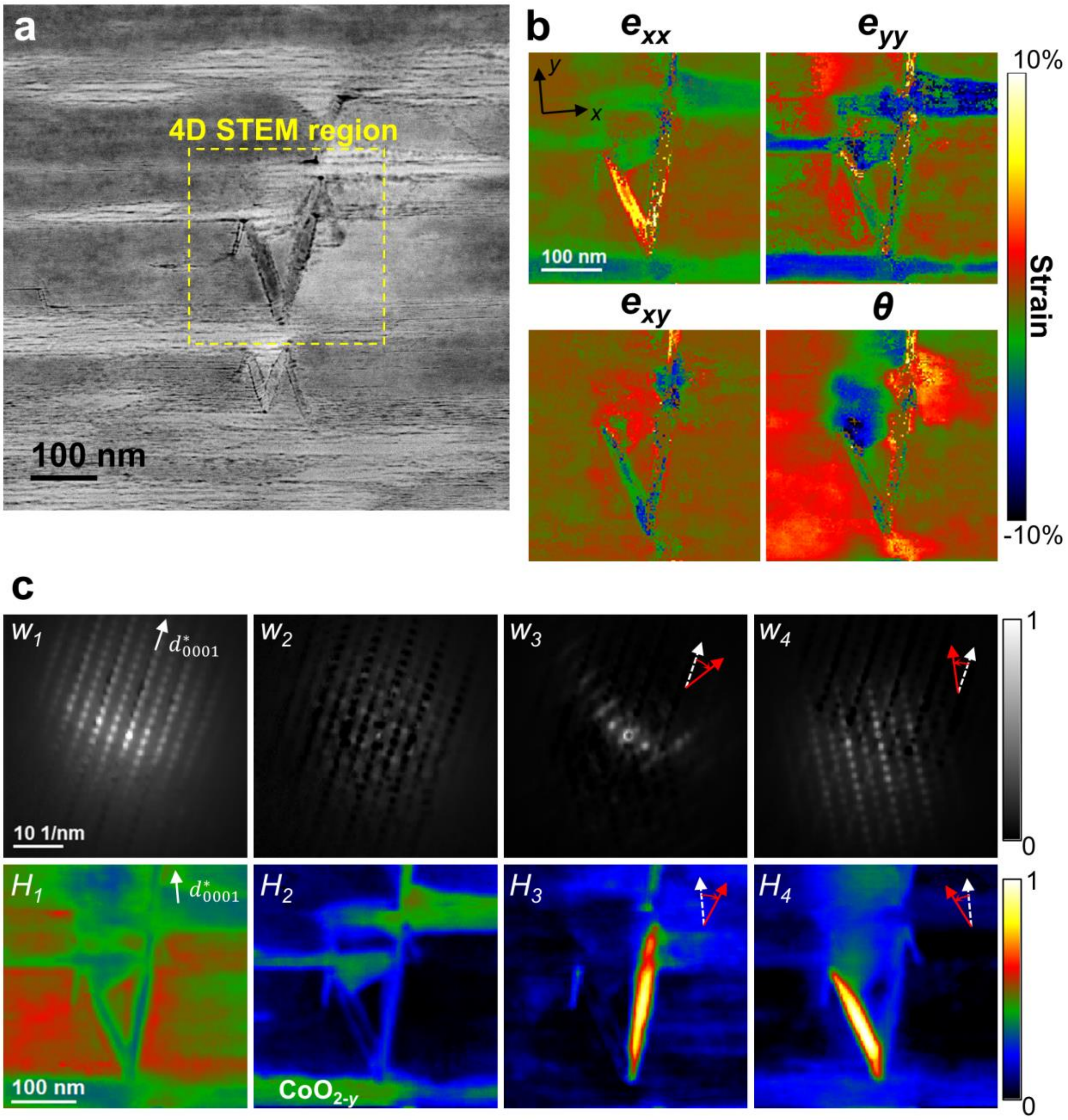

Figure S17. Results of 4D STEM analysis of the other wedge-shaped crack in overcharged lithium cobalt oxide particles. **(a)** HAADF-STEM image of the wedge-shaped crack. 4D STEM is obtained in the region indicated by the yellow box in (a) **(b)** Strain mapping result of the wedge-shaped crack acquired by 4D STEM. The *x* and *y* directions are indicated. **(c)** The first four components (upper row, w_i), and their corresponding coefficient maps (lower row, H_i), of the non-negative matrix factorization result of the 4D STEM data with normalized intensity. The direction of the reciprocal vector d^*_{0001} is indicated.

References

[1] I. Azcarate, W. Yin, C. Méthivier, F. Ribot, C. Laberty-Robert, A. Grimaud, *J. Electrochem. Soc.* **2020**, *167*, 080530.

[2] P. Yan, J. Zheng, M. Gu, J. Xiao, J.-G. Zhang, C.-M. Wang, Nat. Commun. 2017, 8, 14101.

[3] A. Yano, M. Shikano, A. Ueda, H. Sakaebe, Z. Ogumi, J. Electrochem. Soc. 2017, 164, A6116.

[4] J. Kikkawa, S. Terada, A. Gunji, T. Nagai, K. Kurashima, K. Kimoto, *J. Phys. Chem. C* **2015**, *119*, 15823.
